# PTSD as a mediator of the relationship between trauma and psychotic experiences

**DOI:** 10.1017/S0033291720004821

**Published:** 2022-10

**Authors:** Daniela Strelchuk, Gemma Hammerton, Nicola Wiles, Jazz Croft, Katrina Turner, Jonathan Heron, Stanley Zammit

**Affiliations:** 1 Centre for Academic Mental Health, Population Health Sciences, Bristol Medical School, University of Bristol, Bristol, UK; 2 NIHR Biomedical Research Centre at University Hospitals Bristol and Weston NHS Foundation Trust and the University of Bristol, Bristol, UK; 3 MRC Integrative Epidemiology Unit, University of Bristol, Bristol, UK; 4 Centre for Academic Primary Care, Population Health Sciences, Bristol Medical School, University of Bristol, Bristol, UK; 5 Division of Psychological Medicine and Clinical Neuroscience, MRC Centre for Neuropsychiatric Genetics and Genomics, Cardiff University, Cardiff, UK

**Keywords:** Longitudinal, mediation, psychotic experiences, PTSD, trauma

## Abstract

**Background:**

Traumatic experiences are associated with a higher risk of psychotic illnesses, but little is known about potentially modifiable mechanisms underlying this relationship. This study aims to examine whether post-traumatic stress disorder (PTSD) symptoms mediate the relationship between trauma and psychotic experiences (PEs).

**Methods:**

We used data from the Avon Longitudinal Study of Parents and Children to examine whether: PTSD symptoms mediate the relationships between (a) childhood trauma and adolescent PEs (study of adolescent PEs; *n* = 2952), and (b) childhood/adolescent trauma and PEs in early adulthood (study of adult PEs; *n* = 2492). We examined associations between variables using logistic regression, and mediation using the parametric g-computation formula.

**Results:**

Exposure to trauma was associated with increased odds of PEs (adolescent PEs: OR_adjusted_ 1.48, 95% CI 1.23–1.78; adult PEs: OR_adjusted_ 1.57, 95% CI 1.25–1.98) and PTSD symptoms (adolescent PTSD: OR_adjusted_ 1.59, 95% CI 1.31–1.93; adult PTSD: OR_adjusted_ 1.50, 95% CI 1.36–1.65). The association between PTSD symptoms and PE was stronger in adolescence (OR_adjusted_ 4.63, 95% CI 2.34–9.17) than in adulthood (OR_adjusted_ 1.62, 95% CI 0.80–3.25). There was some evidence that PTSD symptoms mediated the relationship between childhood trauma and adolescent PEs (proportion mediated 14%), though evidence of mediation was weaker for adult PEs (proportion mediated 8%).

**Conclusions:**

These findings are consistent with the hypothesis that PTSD symptoms partly mediate the association between trauma exposure and PEs. Targeting PTSD symptoms might help prevent the onset of psychotic outcomes.

## Introduction

Psychotic disorders are some of the most disabling illnesses worldwide (Kyu et al., 2018; Rehm & Shield, 2019). However, current treatment approaches have limited efficacy and a better understanding of the aetiology of psychotic disorders is needed to guide the development of novel targets for intervention.

There is robust evidence that psychosis exists on a continuum (Linscott & Van Os, 2013). Trauma exposure is associated with the development of both psychotic experiences (PEs) in the general population and psychotic disorders in clinical samples, with meta-analyses describing moderate to large associations between childhood adversity and psychosis across this spectrum (Matheson, Shepherd, Pinchbeck, Laurens, & Carr, 2013; Varese, Barkus, & Bentall, 2012). However, less is known about the modifiable pathways through which trauma might contribute to the development of psychosis. A clear understanding of these mechanisms is important because it may allow the identification of targets for interventions to prevent psychosis in people with a history of trauma.

The clearest trauma-related psychopathological outcome is post-traumatic stress disorder (PTSD). PTSD disorder occurs in approximately 10–40% of individuals following exposure to a severe traumatic event (Kessler, Sonnega, Bromet, Hughes, & Nelson, 1995), though symptoms at a sub-diagnostic threshold level are equally common (McLaughlin et al., 2015). Individuals with PTSD have a substantially higher risk of developing schizophrenia and other psychotic disorders (Okkels, Trabjerg, Arendt, & Pedersen, 2017), and PTSD is present, but usually undetected, in approximately 29% of patients in secondary care with a psychotic disorder on screening (Zammit et al., 2018).

It has been proposed that psychotic symptoms could manifest as a more extreme representation of PTSD-like trauma-related psychopathology (Morrison, Frame, & Larkin, 2003). A recent multifactorial model of the role of PTSD in psychosis suggests that unhelpful emotion regulation strategies and high fragmentation of traumatic memories can give rise to intrusions which, depending on their interpretation and coping mechanisms, lead to PEs (Hardy, 2017). For example, the re-experiencing symptoms of PTSD can take the form of hallucinations if intrusions are not recognised as trauma-related and are attributed externally instead (Steel, Fowler, & Holmes, 2005). In addition, dissociation, which is common in PTSD and is associated with childhood trauma in people with psychosis (Rafiq, Campodonico, & Varese, 2018), can deprive the individual from internal and external anchors, increasing vulnerability to impaired reality testing (Allen, Coyne, & Console, 1997) and hallucinations (Moskowitz & Corstens, 2008).

Despite the high prevalence of PTSD in psychosis and plausible explanations for an association, only a few studies have examined the role of PTSD as a mediator in the relationship between trauma and psychosis (Choi et al., 2015; Hardy et al., 2016; McCarthy-Jones, 2018; Murphy, Murphy, & Shevlin, 2015; Peach, Alvarez-Jimenez, Cropper, Sun, & Bendall, 2019; Powers, Fani, Cross, Ressler, & Bradley, 2016; Soosay et al., 2012). Whilst these studies found some evidence for mediation, they were all cross-sectional and therefore unable to determine the direction of relationships, which could have led to biased mediation estimates. Furthermore, concerns about selection bias and confounding in these studies (Williams, Bucci, Berry, & Varese, 2018) highlight that longitudinal studies that can more adequately address these methodological limitations are required to determine whether symptoms of PTSD lie on the causal pathway between trauma and psychosis.

**Fig. 1.**
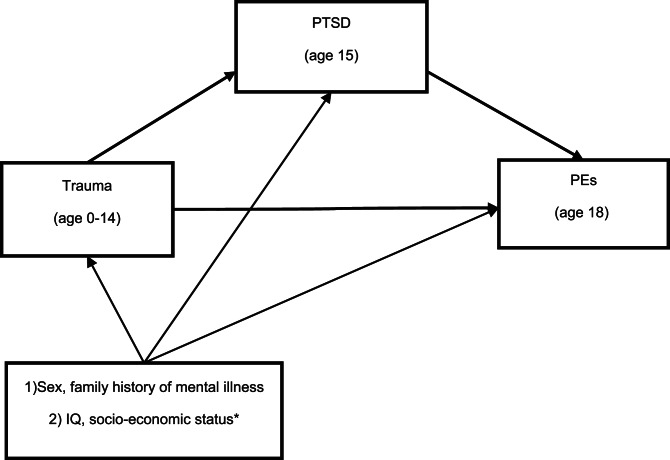
DAG showing the mediation model in the study of adolescent PEs. *Represent the confounders we examined but did not use in final model as they did not change the odds ratios by more than 5%.

**Fig. 2.**
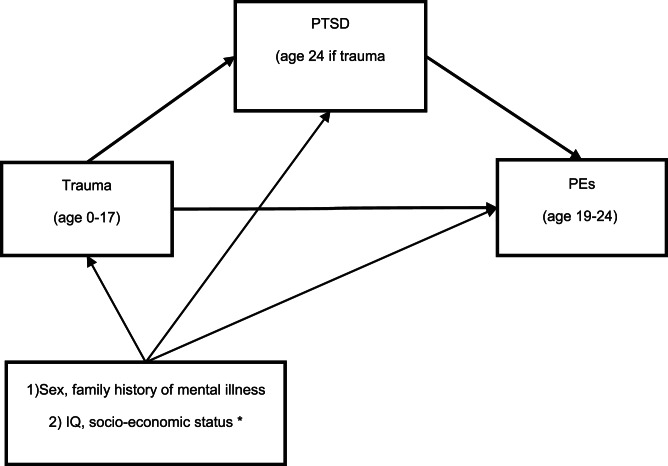
DAG showing the mediation model in the study of adult PEs. *Represent the confounders we examined but did not use in final model as they did not change the odds ratios by more than 5%.

This study therefore aims to examine the extent to which PTSD mediates the relationship between (i) childhood trauma and adolescent PEs, and (ii) childhood/adolescent trauma and PEs in young adulthood (see the directed acyclic graphs in Figs 1 and 2). We examine the role of PTSD symptoms, symptom-clusters and disorder as mediators, and also examine whether mediating effects are the same for hallucinations as for delusions.

## Methods

This study used data from the Avon Longitudinal Study of Parents and Children (ALSPAC) birth cohort study. The original sample consisted of 14062 children born to females living in the south west of England with expected dates of delivery between 1 April 1991 and 31 December 1992 (Boyd et al., 2013; Fraser et al., 2013; Northstone et al., 2019). More details about the ALSPAC study and a fully searchable data dictionary can be found at http://www.bristol.ac.uk/alspac/researchers/our-data/. Informed consent for the use of data collected via questionnaires and clinics was obtained from participants following the recommendations of the ALSPAC Ethics and Law Committee at the time. Study data were collected and managed using the Research Electronic Data Capture (REDCap) tools hosted at the University of Bristol (Harris et al., 2019, 2009). Ethical approval for the study was obtained from the ALSPAC Ethics and Law Committee and the Local Research Ethics Committees.

Of the original sample, 4430 people participated in the assessment of PEs at age 18 (response rate 47%), and 3603 people participated in the assessment at age 24 years (response rate 40%) (see online Supplementary Fig. S1).

### Measures

#### Exposure: trauma

We examined a measure of exposure to trauma over two time periods: (a) the number of trauma-types (physical abuse, emotional abuse, sexual abuse, domestic violence and bullying) that participants experienced between ages 0 and 14 years (when examining the mediating effect of PTSD symptoms at age 15 on adolescent PEs), and (b) the number of trauma types experienced between ages 0 and 17 years (when examining the potential mediating effect of PTSD symptoms at age 24 on adult PEs). These measures were coded from 0 (no trauma exposure) to 5 (exposed to all five trauma types).

Data on exposure to trauma were available from multiple, approximately annual assessments completed by the participants (from ages 7 to 24) and their parents/caregivers (from mother's pregnancy, i.e. pre-birth, up to age 16). All data were reported contemporaneously, except for two questionnaires which asked participants, at age 22 years, about traumatic events experienced in childhood and adolescence to supplement information on sexual abuse, as prior data for this were almost all parent-reported.

#### Mediator: PTSD symptoms

PTSD symptoms at age 15 years were assessed in participants who endorsed having experienced an exceptionally stressful situation, using the Development and Well-Being Assessment (DAWBA) (Goodman, Ford, Richards, Gatward, & Meltzer, 2000) (see online Supplementary Methods 1). The DAWBA has 15 items that assess for symptoms of re-experiencing, avoidance and hypervigilance. Past-month PTSD symptoms at the age of 15 were described by a binary variable (any symptom *v.* none) as the distribution of the variable was skewed.

PTSD symptoms at age 24 years were measured with the Post-traumatic Stress Disorder Checklist for DSM-5 (PCL-5) (Blevins, Weathers, Davis, Witte, & Domino, 2015). The PCL-5 has 20 items which measure four clusters of PTSD as described in DSM-5: re-experiencing, avoidance, negative cognitions and hyperarousal. Participants were asked to identify the worst traumatic event, and then rate their PTSD symptoms in relation to that event (see online Supplementary Methods 2). We derived a binary variable of PTSD symptoms at age 24 years (any symptom *v.* none) in participants for whom the worst traumatic event occurred before the age of 19, so that participants were rating a trauma that occurred prior to the onset of adult PEs (hence we made the assumption that the PTSD symptoms were present since the index trauma, i.e. from before age 19).

For sensitivity analyses, we also derived a binary variable representing a provisional diagnosis of PTSD at age 24 years using a cut-off score of 33 on the PCL-5 (Bovin et al., 2016) in people for whom the self-identified worst traumatic event occurred before the age of 19.

#### Outcome: PEs/psychotic disorder

PEs were assessed at ages 18 (mean age = 17.82, s.d. = 0.46) (Zammit et al., 2013) and 24 years (mean age = 24.03, s.d. = 0.85) (Sullivan et al., 2020) using the semi-structured Psychosis-Like Symptom Interview (PLIKSi), which follows the definitions and rating rules of the Schedule for Clinical Assessment in Neuropsychiatry (SCAN). The PLIKSi assesses hallucinations (auditory and visual), delusions (of being spied on, persecution, thoughts being read, reference, control, grandiose ability, other) and thought disorder (thought broadcasting, thought insertion and thought withdrawal).

Following a series of structured stem questions, interviewers use a semi-structured approach to rate PEs as absent, suspected or definitely present. Examples that clearly meet SCAN definitions have to be provided for an item to be rated as definitely present, and interviewers are trained to ‘rate down’ if uncertain. For the purposes of this study, we only included PEs that were not attributed to the effects of sleep or fever. All interviews were carried out by psychology graduates trained in using the PLIKSi, which has good interrater reliability (*κ* = 0.83).

For the main analyses, we examined the following outcomes: (a) occurrence (Yes/No) of frequent (≥monthly) or distressing (reported as quite or very distressing) PEs between the age of 12 and 18 years (referred to throughout as ‘adolescent PEs’), and (b) incident (new-onset) frequent or distressing PEs between the ages of 19 and 24 years (referred to throughout as ‘adult PEs’). We focused on frequent or distressing PEs as these are more closely related to clinical phenotypes (Legge et al., 2019). We used PEs between the ages of 12 and 18 years rather than 19 and 24 years as the outcome for the adolescent model (see below) as we wanted to capture PEs occurring in closer temporal proximity to the mediator.

As sensitivity analyses (see below), we also examined more stringent measures of past 6-month psychotic disorder at ages 18 and 24 years (defined as having frequent, definite PEs, not attributable to sleep or fever, that had either caused severe distress, had a markedly negative impact on social or occupational functioning, or led to help seeking).

#### Intermediate confounder: self-reported psychotic experiences

These were measured at age 14 years using the PLIKSi-questionnaire, which is a self-report instrument based on the PLIKSi interview but which does not allow for interviewer cross-questioning (Thapar et al., 2012). The questionnaire assesses the presence of visual and auditory hallucinations, beliefs of being spied on, controlled, having one's thoughts read by others or being sent special messages. PEs were coded as present if participants endorsed hallucination experiences as definitely present or delusion experiences as definitely present and having occurred at least monthly over the previous 6 months. This binary variable was used as an intermediate confounder in a sensitivity analysis to minimise potential bias (see ‘Statistical analyses’ below).

#### Baseline confounders

We examined a number of variables as potential confounders as they have been shown to be associated with trauma, PTSD and PEs in other studies and could potentially confound the relationships of interest in our study. These included sex, IQ (assessed at age 8 using the WISC), social class (classes I–V as defined by the UK Registrar General's occupational coding based on parents’ occupation) and family history of mental illness during early childhood (assessed as parental history of schizophrenia, anorexia, bulimia, alcoholism, attempted suicide or anxiety/depression). As including all potential confounders in the multivariable models led to a substantial reduction in sample size due to missing data, we examined the effect of individually adjusting for each confounder, and only retained in the final model variables that changed the unadjusted odds ratios by more than 5%. When examining associations between PTSD and PEs, we additionally adjusted for exposure to trauma.

## Statistical analyses

All analyses were carried out in Stata version 15. We examined (i) the association between exposure to trauma between ages 0 and 14 and frequent or distressing PEs between ages 12 and 18, and the proportion of this that was mediated by PTSD symptoms at age 15, and (ii) the association between exposure to trauma between ages 0 and 17 and incident frequent or distressing PEs between ages 19 and 24, and the proportion of this that was mediated by PTSD symptoms at age 24. We used logistic regression to examine the association between trauma and PTSD, PTSD and PEs, and trauma and PEs. Odds ratios (OR), 95% confidence intervals (95% CI) and *p* values are reported.

We tested for mediation of the trauma–PEs relationship by PTSD within a counterfactual framework using the parametric g-computation formula (see online Supplementary Methods 3 for a definition of causal effects in the counterfactual approach) (Daniel, de Stavola, & Cousens, 2011). G-computation involves estimating the g-computation formula using Monte Carlo simulation to simulate the mediator, outcome and intermediate confounder under each hypothetical intervention scenario (i.e. the mediator is simulated when the exposure takes both values 0 and 1). G-computation was implemented using the STATA package ‘gformula’ (Daniel et al., 2011). The confidence intervals were calculated using 1000 bootstraps.

### Sensitivity analyses

In sensitivity analyses, we: (i) examined a narrower definition of psychotic outcome (psychotic disorder) to see whether results were stronger for a more clinically-relevant outcome; (ii) included a measure of PEs at age 14 years as an intermediate confounder in the mediation model for adolescent PEs because it was possible that the PTSD symptoms at age 15 occurred as a consequence of PEs present before this age (see online Supplementary Fig. S2); (iii) repeated analyses using imputation to address potential bias due to missing data; (iv) examined whether specific PTSD clusters (re-experiencing, hyper-arousal, avoidance and negative beliefs) played a more important mediation role than others; (v) used PTSD diagnosis rather than the presence of any PTSD symptom as a mediator for the adult PEs model as a more stringent measure of PTSD symptomatology; and (vi) compared the mediating effect of PTSD symptoms on hallucinations with that on delusions.

### Missing data

To address the problem of missing data, we used multiple imputation by chained equations (using the ‘ice’ command in STATA). The exposure, mediator and baseline confounders were imputed up to the sample size of complete outcome (*n* = 4430 in adolescent PEs study; *n* = 3603 in adult PEs study). We created 50 imputed datasets using all variables in the analysis and 11 auxiliary variables that predicted missingness and were associated with the variables of interest to make the missing at random assumption more plausible (see online Supplementary Methods 4).

As it was not possible to run mediation models in multiply imputed datasets using the ‘gformula’ STATA package, we used the inbuilt ‘gformula’ command to impute data and test for mediation simultaneously (see online Supplementary Methods 5 for more detail).

## Results

### Study sample

The total number of participants with complete data on exposure, outcome, mediator and confounding variables for the adolescent PEs model was 2952, and for the adult PEs model was 2492. Participants who were included in the adolescent model compared with those who were excluded (*n* = 10 991) were more likely to be female, to come from a family with a higher social class, and less likely to report a family history of mental health disorder (see Table 1), and sample representativeness was similar for the adult model sample. As individually-adjusting for IQ and socio-economic status made minimal difference to the estimates in any of the models, we only included sex and family history of mental health disorder as confounders in our adjusted models.

**Table 1.** Study of adolescent PE: sample characteristics of participants included and excluded from the study

	Included	Excluded	OR (95% CI)	*p* value
Female sex	1706/2952 (57.8)	5040/10 991 (45.9)	1.62 (1.49–1.76)	<0.001
Number of trauma types (ages 0–14)				
0	1599/2952 (54.2)	4461/7840 (56.9)	0.36 (0.34–0.38)	0.01
1	830 (28.1)	2157 (27.5)	1.07 (0.97–1.18)	
2	359 (12.2)	814 (10.4)	1.23 (1.07–1.41)	
3	133 (4.5)	348 (4.4)	1.07 (0.87–1.31)	
4	26 (0.9)	58 (0.7)	1.25 (0.78–1.99)	
5	5 (0.2)	<5 (0.03)	6.97 (1.35–35.98)	
PTSD symptoms (age 15)	75/2952 (2.5)	71/2137 (3.3)	0.76 (0.55–1.05)	0.10
Frequent or distressing PEs (age 12–18)	94/2952 (3.2)	86/1478 (5.8)	0.53 (0.39–0.72)	<0.001
Parental psychiatric history	469/2952 (15.9)	1929/10 157 (19.0)	0.81 (0.72–0.90)	<0.001
Mean IQ	108.9 (*n* = 2723)	101.2 (*n* = 4306)	1.03 (1.02–1.03)	<0.001
Parents’ highest social class	572/2829 (20.2)	951/8642 (11.0)	1.60 (1.51–1.69)	<0.001
Lowest income	322/2733 (11.8)	1667/7180 (23.2)	1.28 (1.24–1.33)	<0.001
Maternal education: <O levels	448/2916 (15.4)	3274/9469 (34.6)	1.91 (1.80–2.01)	<0.001
Parental dug use	233/2942 (8.1)	1073/10 471 (10.3)	0.76 (0.65–0.87)	<0.001

### Adolescent PEs model

In total, 1353 individuals in our sample (46%) experienced at least one type of trauma between ages 0 and 14 years, 75 (2.5%) had 1 or more PTSD symptoms at age 15 years, and 94 (around 3%) reported distressing or frequent PEs when assessed at age 18 (see Table 1 and online Supplementary material S1 for more detail).

Trauma exposure was associated with increased odds of both PEs (OR_adjusted_ 1.48, 95% CI 1.23–1.78, *p* < 0.001) and PTSD symptoms (OR_adjusted_ 1.59, 95% CI 1.31–1.93, *p* < 0.001) (Table 2). There was a strong association between PTSD symptoms and PEs, and this slightly attenuated after adjusting for confounding (OR_crude_ 6.49, 95% CI 3.37–12.50; OR_adjusted_ 4.63, 95% CI 2.34–9.17, *p* < 0.001).

**Table 2.** Associations between trauma, PTSD symptoms and frequent or distressing PE^a^

Exposure	Outcome	Unadjusted			Adjusted^b^		
		OR	95% CI	*p* value	OR	95% CI	*p* value
Trauma (ages 0–14)	PEs (age 12–18)	1.48	1.23–1.77	<0.001	1.48	1.23–1.78	<0.001
Trauma (ages 0–17)	PEs (ages 19–24)	1.56	1.24–1.96	<0.001	1.57	1.25–1.98	<0.001
Trauma (ages 0–14)	PTSD symptoms (age 15)	1.69	1.40–2.09	<0.001	1.59	1.31–1.93	<0.001
Trauma (ages 0–17)	PTSD symptoms (age 24)	1.49	1.36–1.64	<0.001	1.50	1.36–1.65	<0.001
PTSD symptoms (age 15)	PEs (age 12–18)	6.49	3.37–12.50	<0.001	4.63	2.34–9.17	<0.001
PTSD symptoms (age 24)	PEs (ages 19–24)	2.07	1.06–4.04	0.03	1.62	0.80–3.25	0.18

a Study of adolescent PEs, *N* = 2952; study of adult PEs, *N* = 2492.

b Sex and family history of mental illness adjusted for in all models; exposure to trauma additionally adjusted for in PTSD-PEs regressions.

There was some evidence that PTSD symptoms mediated the relationship between trauma and PEs (natural indirect effect OR_adjusted_ 1.05, 95% CI 1.01–1.10). The proportion mediated in the adjusted analysis was approximately 14% (Table 3).

**Table 3.** Total, direct and indirect effects for frequent or distressing PEs

	PEs (12–18)^a^						PEs (19–24)^b^					
	Unadjusted			Adjusted^c^			Unadjusted			Adjusted^c^		
	OR	95% CI	*p*	OR	95% CI	*p*	OR	95% CI	*p*	OR	95% CI	*p*
Total causal effect	1.48	1.26–1.73	<0.001	1.48	1.25–1.75	<0.001	1.56	1.24–1.88	<0.001	1.57	1.28–1.92	<0.001
Natural direct effect	1.38	1.17–1.63		1.40	1.18–1.67		1.51	1.20–1.80		1.51	1.24–1.84	
Natural indirect effect	1.07	1.02–1.12		1.05	1.01–1.10		1.03	0.98–1.09		1.03	0.98–1.09	
Proportion mediated	17%			14%			8%			8%		

a Study of adolescent PEs, *N* = 2952.

b Study of adult PEs, *N* = 2492.

c Confounders adjusted for: sex and family history of mental health illness.

### Adult PEs model

The frequency of PTSD symptoms was much higher at age 24 years (around 15%) compared with age 15 years (2.5%), and 45 individuals (1.8%) were rated as having incident distressing or frequent PEs between ages 19 and 24 years (online Supplementary material S2 for more detail).

There was strong evidence of an association between childhood/adolescent exposure to trauma and both PEs (OR_adjusted_ 1.57, 95% CI 1.25–1.98, *p* < 0.001) and PTSD symptoms (OR_adjusted_ 1.50, 95% CI 1.36–1.65, *p* < 0.001) at age 24. There was weaker evidence of an association between PTSD symptoms and PEs at this age after adjusting for childhood/adolescent trauma (OR_adjusted_ 1.62, 95% CI 0.80–3.25, *p* = 0.18) (Table 2).

There was weak evidence that PTSD symptoms mediated the association between trauma and adult PEs, with the 95% CIs including the null value (natural indirect effect: OR_adjusted_ 1.03, 95% CI 0.98–1.09) (Table 3). The proportion mediated was approximately 8%.

### Sensitivity analyses

First, results for adolescent psychotic disorder (online Supplementary material S3) were stronger than those from our main analyses, with approximately 19% of the association between trauma and psychotic disorder being mediated by PTSD symptoms. However, the confidence intervals for psychotic disorder and PEs substantially overlapped. It was not possible to test for mediation for adult psychotic disorder as there was little evidence of an association between PTSD symptoms and disorder at age 24.

Second, we included a measure of PEs at age 14 years as an intermediate confounder in the mediation model for adolescent PEs to minimise the potential that PTSD symptoms at age 15 occurred as a consequence of PEs present before this age. The proportion of the association between exposure to trauma and PEs that was mediated by PTSD symptoms was very similar in these analyses to the findings from the primary analysis (13% compared with 14% in the latter) (online Supplementary material S4).

Third, we examined whether our results were likely to be affected by selection bias as a result of missing data and found that results including imputed data were very similar to those using complete data (online Supplementary material S5).

Fourth, when we examined the role of individual PTSD symptom-clusters as mediators, the proportion mediated showed some variation (ranging from 4% for re-experiencing mediating effects on adult PEs to 15% for avoidance symptoms mediating effects on adolescent PEs), but confidence intervals around the natural indirect effect (NIE) for each symptom-cluster overlapped substantially, providing little evidence of cluster-specific effects (online Supplementary material S6).

Fifth, the proportion mediated by PTSD diagnosis in young adulthood was greater than that mediated by any PTSD symptom (12% compared with 8%) (online Supplementary material S7).

Finally, in the study of adolescent PEs, the proportion mediated by PTSD symptoms was similar for hallucinations and delusions (12% and 15%) (online Supplementary material S8). In the study of adult PEs, the number of people with delusions was too low to enable us to test for mediation and to compare this with hallucinations at this age (online Supplementary material S8).

## Discussion

To our knowledge, this is the first longitudinal study to investigate the role of PTSD as a mediator of the relationship between trauma and PEs. Exposure to trauma was associated with an increased odds of PEs and PTSD symptoms, both in adolescence and early adulthood. The association between PTSD symptoms and PEs was stronger in adolescence than in adulthood. Our mediation analyses provide some support for the role of PTSD symptoms partially mediating the relationship between childhood trauma and adolescent PEs, although the evidence of PTSD mediating the effect on adult PEs was weaker. Findings from our sensitivity analyses show that the proportion mediated by PTSD symptoms increased when examining more narrowly defined outcomes (14% for adolescent PEs compared to 19% for psychotic disorder), though the confidence intervals overlapped. There was little evidence to support the hypothesis that certain PTSD clusters had a more important role in mediation than others, or that PTSD symptoms played a more important role in the development of hallucinations than delusions.

Our study has some important strengths including comprehensive measures of trauma exposure assessed prospectively prior to our outcome measures, use of semi-structured interviews to assess psychotic outcomes, the use of robust statistical methods to test for mediation and a rigorous approach to examine non-causal explanations for any associations observed. However, our results need to be interpreted in the context of a number of limitations.

First, whilst our estimates remained relatively unchanged after adjustment, residual confounding could still be present, as for any observational study. Second, although assessments of trauma, PTSD and PEs in adolescence were temporally ordered, there was some overlap between the measures of PTSD and PEs. We therefore modelled self-reported PEs at age 14 as an intermediate confounder in a sensitivity analysis, and although results were very similar to those from the main analysis, we cannot be certain that our mediation estimates are unbiased. As our measures of PTSD and PEs in adulthood were collected contemporaneously, the temporal order of PTSD and PEs in this analysis could not be clearly determined, and therefore the measure of PTSD was only rated for traumatic events that participants said occurred prior to the onset of their PEs, to minimise potential bias.

Third, as in most longitudinal studies, there was substantial attrition over time. Results were very similar after multiple imputation using auxiliary variables to make the missing at random assumption more plausible, suggesting that selection bias is unlikely to explain our results, though the possibility cannot be excluded.

Fourth, although we used validated measures of PTSD, we were unable to use symptom dose as a mediator due to statistical modelling limitations. This may have led to information loss and hence we might have underestimated the mediating effect of PTSD symptoms. Furthermore, collapsing symptom data into a binary measure makes it unclear whether the mediation effect operates through symptoms specific to PTSD, or through symptoms such as sleep problems that index psychopathology more broadly. To address this limitation, we examined PTSD diagnosis and PTSD symptom-clusters as mediators and results were consistent, suggesting that this latter explanation is unlikely.

Finally, although raters were trained to cross-question in depth during the semi-structured interview to ensure PEs met SCAN criteria, it is possible that measurement error still exists, for example, if interviewers mistakenly rated intrusive memories as PEs.

Other studies have also reported evidence consistent with the mediation effects of PTSD (Alameda et al., 2020; Williams et al., 2018). In general, the proportion mediated reported by these studies is substantially greater than we observed in our sample [34% and 45% in the only two studies that reported values for this (Choi et al., 2015; Soosay et al., 2012)]. However, all these studies were cross-sectional, and a temporal relationship between variables could not be established. Thus, if PTSD symptoms developed as a direct result of the psychotic phenomena experienced, or in response to involuntary detainment (Morrison et al., 2003), estimates of mediation may have been biased and over-inflated.

Moreover, few of these prior studies adequately controlled for confounding, and some used self-report measures of PEs which may have conflated psychotic and PTSD symptoms. Our findings, using a longitudinal design, adjusting for a range of confounders and using semi-structured interview measures of PEs, suggest that only a relatively small proportion of the relationship between trauma exposure and PEs is mediated by PTSD.

Whilst we found stronger evidence for PTSD mediating the effect of trauma on adolescent PEs than on adult ones, the confidence intervals for these analyses overlapped substantially, providing little evidence of age-specific effects. There is also little evidence that childhood represents a more sensitive period of risk than adolescence for trauma exposure in relation to psychosis (Croft et al., 2019), although less fully-developed neural systems at younger ages may lead to increased stress sensitivity and greater difficulty in contextual integration of traumatic experiences (Steel, 2015). This could result in increased levels of dissociation and a greater difficulty in determining whether intrusive experiences are trauma-related memories or originate as external to the self (e.g. hallucinations). Furthermore, dissociation, whilst being an important coping mechanism in dealing with inescapable threat, is more likely to occur following trauma in childhood, and is associated with trauma-related intrusive memories, poor contextual processing and an increased risk of psychotic outcomes (Laposa & Alden, 2008; Steel, 2015; Varese et al., 2012). Hence it is plausible that the relationship between trauma, PTSD and PEs is more clearly delineated during adolescence than in adulthood, although there is little evidence to support this theory at present. Indeed, the only prospective study to date of dissociation found no evidence for it mediating the trauma–psychosis relationship (Thompson et al., 2016).

Our results are consistent with the hypothesis that trauma exposure has a causal role in the development of psychosis and that, for some people, PEs might reflect more extreme expressions of symptoms that characterise PTSD. It has been proposed that dysfunctional emotion regulation strategies such as hyperarousal, avoidance or dissociation are vulnerability factors for the occurrence of anomalous experiences and trauma-related intrusions which, depending on their interpretation, could give rise to PEs (Hardy, 2017). For example, a state of hyperarousal can increase the likelihood of noticing threatening stimuli, and avoidance and dissociation can lead to inadequate processing of traumatic memories which in turn increases the likelihood of experiencing trauma-related intrusions. If these intrusions are not recognised as being part of the trauma but are attributed externally, they may be regarded as new perceptions (Steel et al., 2005). Furthermore, the negative cognitions about the self, others and the world can make people feel vulnerable and more likely to appraise innocuous events in a threatening manner, whilst voidance prevents people from testing the validity of their interpretations.

Other psychological mechanisms that are hypothesised to mediate the association between trauma and PEs include insecure attachment and avoidance, depression and anxiety, and dysfunctional emotion regulation strategies (Williams et al., 2018). The extent to which any of these mechanisms have mediating effects independent from those of PTSD symptoms is yet to be determined, although these constructs all overlap to some extent.

Although the percentage mediated by PTSD symptoms is quite modest, the role of PTSD in the development of psychosis warrants further investigation given the poor outcome of psychotic illnesses and the existence of effective treatments for PTSD (Bisson, Roberts, Andrew, Cooper, & Lewis, 2013). Trauma-focused therapies facilitate the reconsolidation of traumatic memories, allowing them to be adequately processed and contextually integrated. As dissociation and inadequate trauma processing play a key role in the occurrence of trauma-related intrusions and have been hypothesised to have a similar role in PEs, it is theoretically possible that targeting the inadequate processing of a traumatic event using trauma-focused therapies will also lead to reduced levels of PEs. Indeed, there is preliminary evidence to support this hypothesis (de Bont et al., 2016; van den Berg et al., 2015) and our findings suggest that further trials are warranted.

Our findings need to be interpreted in the context of the limitations described above but are consistent with the hypothesis that PTSD symptoms partly mediate the relationship between exposure to trauma and development of PEs. Given that treatment for PTSD is highly effective, our study indicates that processing of disturbing traumatic memories may help prevent the occurrence of PEs and transition to psychotic disorder.

## Supplementary material

For supplementary material accompanying this paper visit https://doi.org/10.1017/S0033291720004821. click here to view supplementary material
