# Supplementary material for: PTSD as a mediator of the relationship between trauma and psychotic experiences
Source: Psychol Med. 2020 Dec 14;52(13):2722–30. doi: 10.1017/S0033291720004821 (PMC9647519; doi:10.1017/S0033291720004821)
Supplement: Supplementary file 1 [file S0033291720004821sup.zip › S0033291720004821sup001.docx]

**Supplement Figure 1**. Flowchart showing complete data on variables of interest for study of adolescent PEs

Original sample size

N=13,943

PEs (age18)

N=4,430

Complete case

N=2,952

PTSD (age 15)

N=5,089

Trauma (age 0-14)

N=10,792

Baseline confounders

N=13,109

Intermediate confounder

(age 14)

N=5,718
