# Supplementary material for: PTSD as a mediator of the relationship between trauma and psychotic experiences
Source: Psychol Med. 2020 Dec 14;52(13):2722–30. doi: 10.1017/S0033291720004821 (PMC9647519; doi:10.1017/S0033291720004821)
Supplement: Supplementary file 1 [file S0033291720004821sup.zip › S0033291720004821sup002.docx]

**Supplement Figure 2**. DAG showing the mediation model in study of adolescent PEs

with self-report PEs at age 14 as an intermediate confounder

Trauma

(age 0-14)

PEs

(age 18)

Self-report PEs

(age 14)

PTSD

(age 15)

1)Sex

2)Family history of mental illness
