# Supplementary material for: PTSD as a mediator of the relationship between trauma and psychotic experiences
Source: Psychol Med. 2020 Dec 14;52(13):2722–30. doi: 10.1017/S0033291720004821 (PMC9647519; doi:10.1017/S0033291720004821)
Supplement: Supplementary file 1 [file S0033291720004821sup.zip › S0033291720004821sup003.docx]

**Supplement Methods 1**

PTSD symptoms at age 15 years were assessed with the DAWBA. Those participants who endorsed having experienced an exceptionally stressful situation were then asked whether those experiences were affecting their current behaviour, feelings and concentration. Those who said “Yes” were asked to rate the severity of their PTSD symptoms over the previous 4 weeks on a scale from 1 to 3 where 1 represented “No”, 2 “A little” and 3 “A lot”.

**Supplement Methods 2**

PTSD symptoms at age 24 years were measured with the Post-traumatic Stress Disorder Checklist for DSM-5 (PCL-5). Responders rated their symptoms on a 5 point scale (0 “Not at all”, 1 “A little bit”, 2 “Moderately”, 3 ”Quite a bit”, 4 “Extremely”).

**Supplement Methods 3**

In a counterfactual approach, the total causal effect (TCE) is the value that the outcome would take if everybody had been exposed to trauma compared with no one having trauma. The natural direct effect (NDE) is modelled as the direct effect of exposure X = 1 (exposed to trauma) versus the absence of exposure X = 0 (not exposed to trauma) on outcome Y (psychotic experiences) if mediator m (PTSD symptoms) were set to whatever it would be for X = 0. The natural indirect effect (NIE) is modelled as the effect on outcome Y if the exposure were fixed at X = 1 and mediator m were changed from the level it would take if X = 0 to the level it would take if X = 1.

**Supplement Methods 4**

The auxilliary variables that were used to make the missing at random assumption more plausible were: trauma as reported at ages 23 and 24 years, PTSD as reported at age 24 using clinician and self-report measures, any ICD-10 or DSM-IV diagnosis at age 10 and 13 years, other emotional and behavioural difficulties, drug use, alcohol use, IQ and parents’ social class. We used predictive mean matching to deal with non-normally distributed variables.

**Supplement Methods 5**

For building the imputation model in ‘gformula’, we used the same variables and set up the same prediction equations that we used in ICE. G-formula uses single imputation (i.e. it draws one improper imputation for each missing value) and minimises over-precision of the standard errors via bootstrapping, not analytically as when using multiple imputation by chained equations (Daniel, de Stavola and Cousens, 2011).

S1. Frequency of the number of trauma types (age 0-14 years) in participants with PTSD (age 15 years) and PEs (age 12-18 years)*

|  | PTSD symptoms | | | PEs | | | Psychotic disorder | |
| --- | --- | --- | --- | --- | --- | --- | --- | --- |
| Number of trauma types | No (%) | Yes (%) | No (%) | | Yes (%) | No (%) | | Yes (%) |
| 0 | 1.575 (98.5) | 24 (1.5) | 1.571 (98.2) | | 28 (1.8) | 1.593 (99.6) | | 6 (0.4) |
| 1 | 809 (97.5) | 21 (2.5) | 792 (95.4) | | 38 (4.6) | 821 (98.9) | | 9 (1.1) |
| 2 | 340 (94.7) | 19 (5.3) | 340 (94.7) | | 19 (5.3) | 354 (98.6) | | 5 (1.4) |
| 3 | 125 (94.0) | 8 (6.0) | 125 (94.0) | | 8 (6.0) | 129 (97.0) | | 4 (3.0) |
| 4 | 25 (96.2) | 1 (3.8) | 25 (96.2) | | 1 (3.8) | 25 (96.2) | | 1 (3.8) |
| 5 | 3 (60.0) | 2 (40.0) | 5 (100.0) | | 0 (0.0) | 5 (100) | | 0 (0.0) |
|  |  |  | PEs | | | Psychotic disorder | | |
| PTSD symptoms |  |  | No (%) | | Yes (%) | No (%) | | Yes (%) |
| 0 |  |  | 2.795 (97.2) | | 82 (2.8) | 2.858 (99.3) | | 19 (0.7) |
| 1 |  |  | 63 (84.0) | | 12 (16.0) | 69 (92.0) | | 6 (8.0) |

*N=2,952

S2. Frequency of the number of trauma types (age 0-17 years) in participants with PTSD (assessed at age 24 years) and psychotic outcomes

(age 19-24 years)*

|  | PTSD symptoms | | PEs | | Psychotic disorder | |
| --- | --- | --- | --- | --- | --- | --- |
| Number of trauma types | No (%) | Yes (%) | No (%) | Yes (%) | No (%) | Yes (%) |
| 0 | 1,066 (89.4) | 126 (10.6) | 1,182 (99.2) | 10 (0.8) | 1,190 (99.8) | 2 (0.2) |
| 1 | 583 (85.0) | 103 (15.0) | 672 (98.0) | 14 (2.0) | 683 (99.6) | 3 (0.4) |
| 2 | 284 (80.7) | 68 (5.3) | 342 (97.2) | 10 (2.8) | 347 (98.6) | 5 (1.4) |
| 3 | 147 (71.4) | 59 (28.6) | 196 (95.2) | 10 (4.8) | 200 (97.1) | 6 (2.9) |
| 4 | 31 (62.0) | 19 (38.0) | 50 (100) | 0 (0.00) | 49 (98.0) | 1 (2.0) |
| 5 | 3 (50.0) | 3 (50.0) | 5 (83.3) | 1 (16.7) | 6 (100) | 0 (0.0) |
|  |  |  | PEs | | Psychotic disorder | |
| PTSD symptoms |  |  | No (%) | Yes (%) | No (%) | Yes (%) |
| 0 |  |  | 2,081 (85.0) | 366 (14.9) | 2,101 (84.9) | 374 (15.1) |
| 1 |  |  | 33 (73.3) | 12 (26.7) | 13 (76.5) | 4 (23.5) |
| PTSD diagnosis |  |  | No (%) | Yes (%) | No (%) | Yes (%) |
| 0 |  |  | 2,386 (97.5) | 61 (2.5) | 2,412 (97.4) | 63 (2.6) |
| 1 |  |  | 40 (88.9) | 5 (11.1) | 14 (82.3) | 3 (17.7) |

*N=2.492

S3. Associations between trauma, PTSD symptoms and *psychotic disorder**. Total, direct and indirect effects for *psychotic disorder*.

| Exposure | Outcome | **Unadjusted** | | | | | | | **Adjusted**** | | | | | |
| --- | --- | --- | --- | --- | --- | --- | --- | --- | --- | --- | --- | --- | --- | --- |
|  |  | OR | | 95%CI | | P value | | OR | | | 95%CI | | P value | |
| Trauma  (ages 0-14) | Psychotic disorder  (age 18) | 1.79 | | 1.30  2.45 | | <0.001 | | 1.85 | | | 1.35 2.53 | | <0.001 | |
| Trauma  (ages 0-17) | Psychotic disorder  (age 24) | 2.07 | | 1.46 2.94 | | 0.001 | | 2.02 | | | 1.41 2.88 | | <0.001 | |
| PTSD symptoms  (age 15) | Psychotic disorder  (age 18) | 13.08 | | 5.07  33.77 | | <0.001 | | 8.55 | | | 3.13 23.33 | | <0.001 | |
| PTSD symptoms  (age 24) | Psychotic disorder  (age 24) | 1.73 | | 0.56  5.33 | | 0.34 | | 1.03 | | | 0.31 3.35 | | 0.96 | |
|  | | | | | | | | | | | | | | |
|  | | | **Psychotic disorder (age 18)** | | | | | | | | | | | |
|  |  |  | **Unadjusted** | | | | | | | **Adjusted**** | | | | |
|  |  |  | OR | | 95% CI | | p | | OR | | | 95% CI | | p |
| Total causal effect | | | 1.80 | | 1.33 2.42 | | <0.001 | | 1.88 | | | 1.28 2.76 | | 0.001 |
| Natural direct effect | | | 1.57 | | 1.12 2.21 | |  | | 1.67 | | | 1.11 2.49 | |  |
| Natural indirect effect | | | 1.14 | | 1.03 1.27 | |  |  | 1.13 | | | 1.02 1.24 | |  |
| Proportion mediated | | | 23% | | | | | | | 19% | | | | |

^*^N=2,952; **Sex and family history of mental illness adjusted for in all models; Exposure to trauma additionally adjusted for in PTSD-psychotic disorder regressions

S4. Total, direct and indirect effects for study of adolescent PEs. Frequent and distressing experiences at age 14 as an intermediate confounder*

|  | **PEs (age 12-18)**** | | | | | |
| --- | --- | --- | --- | --- | --- | --- |
|  | **Unadjusted** | | | **Adjusted***** | | |
|  | OR | 95% CI | p | OR | 95% CI | p |
| Total causal effect | 1.47 | 1.25 1.73 | <0.001 | 1.48 | 1.23 1.76 | <0.001 |
| Natural direct effect | 1.38 | 1.15 1.64 |  | 1.40 | 1.17 1.69 |  |
| Natural indirect effect | 1.07 | 1.01 1.13 |  | 1.05 | 1.00 1.10 |  |
| Proportion mediated | 17% | | | 13% | | |

^*^In regression analyses, the association between PTSD symptoms and PEs after adjusting for psychotic-like experiences at age 14 in addition to sex and family history of mental health illness was OR = 3.66, 95%CI 1.75-7.68, p=0.001; **N=2,952; *** Baseline confounders adjusted for in mediation model: sex and family history of mental illness.

S5. Imputed analyses - Associations between trauma, PTSD symptoms and PEs*. Total, direct and indirect effects for PEs

| **Exposure** | **Outcome** | **Unadjusted** | | | | | | | | | | | **Adjusted**** | | | | | | | | |
| --- | --- | --- | --- | --- | --- | --- | --- | --- | --- | --- | --- | --- | --- | --- | --- | --- | --- | --- | --- | --- | --- |
|  |  | **OR** | | | **95%CI** | | | | **P value** | | | **OR** | | | **95%CI** | | | | | **P value** | |
| Trauma  (ages 0-14) | PEs  (age 18) | 1.54 | | | 1.28  1.85 | | | | <0.001 | | | 1.51 | | | 1.25 1.81 | | | | | <0.001 | |
| Trauma  (ages 0-17) | PEs  (ages 19-24) | 1.54 | | | 1.29  1.84 | | | | <0.001 | | | 1.51 | | | 1.25 1.81 | | | | | <0.001 | |
| Trauma  (ages 0-14) | PTSD symptoms  (age 15) | 1.74 | | | 1.46   2.06 | | | | <0.001 | | | 1.62 | | | 1.36 1.94 | | | | | <0.001 | |
| Trauma  (ages 0-17) | PTSD symptoms  (age 24) | 1.48 | | | 1.34   1.63 | | | | <0.001 | | | 1.48 | | | 1.35 1.64 | | | | | <0.001 | |
| PTSD symptoms  (age 15) | PEs  (age 18) | 6.93 | | | 4.02 11.97 | | | | <0.001 | | | 4.82 | | | 2.69 8.62 | | | | | <0.001 | |
| PTSD symptoms  (age 24) | PEs  (ages 19-24) | 2.07 | | | 1.06  4.01 | | | | 0.03 | | | 1.74 | | | 0.86 3.53 | | | | | 0.12 | |
|  | | | | | | | | | | | | | | | | | | | | | |
|  | | **PEs (age 12-18)** | | | | | | | | | | | **PEs (age 19-24)** | | | | | | | | |
|  |  | Unadjusted | | | | | Adjusted | | | | | | Unadjusted | | | | | Adjusted | | | |
|  |  | OR | 95% CI | p | | OR | | 95% CI | |  | OR | | | 95% CI | | p | OR | | 95% CI | | p |
| Total causal effect | | 1.40 | 1.23 1.58 | <0.001 | | 1.35 | | 1.19 1.54 | | <0.001 | 1.46 | | | 1.22 1.76 | | <0.001 | 1.44 | | 1.19 1.74 | | <0.001 |
| Natural direct effect | | 1.31 | 1.16 1.49 |  | | 1.30 | | 1.14 1.48 | |  | 1.42 | | | 1.18 1.71 | |  | 1.40 | | 1.15 1.69 | |  |
| Natural indirect effect | | 1.06 | 1.01 1.12 |  |  | 1.04 | | 1.00 1.09 | |  | 1.03 | | | 0.98 1.09 | |  | 1.03 | | 0.97 1.09 | |  |
| Proportion mediated | | 18% | | | | | 15% | | | | | | 8% | | | | | 8% | | | |

*Study of adolescent PEs, N=4,430; Study of adult PEs, N=3,603; **Sex and family history of mental illness adjusted for in all models; Exposure to trauma additionally adjusted for in PTSD-PEs regressions

S6. Total, direct and indirect effects for frequent or distressing PEs – Mediators: Re-experiencing, avoidance, hyper-arousal, negative beliefs

|  | **Outcome: PEs (12-18)*** | | | | | | | | | **Outcome: PEs (19-24)**** | | | | | | | | | | | |
| --- | --- | --- | --- | --- | --- | --- | --- | --- | --- | --- | --- | --- | --- | --- | --- | --- | --- | --- | --- | --- | --- |
|  | **Unadjusted** | | | | **Adjusted****** | | | | **Unadjusted** | | | | | | | | **Adjusted****** | | | | |
|  | **OR** | **95% CI** | **p** | **OR** | | **95% CI** | **p** | **OR** | | | **95% CI** | | **p** | | **OR** | | | **95% CI** | | **p** | |
|  | **Mediator: Re-experiencing** | | | | | | | | | **Mediator: Re-experiencing** | | | | | | | | | | | |
| Total causal effect | 1.48 | 1.26 1.73 | <0.001 | 1.47 | | 1.24 1.74 | <0.001 | 1.56 | | | 1.28 1.89 | | <0.001 | | 1.57 | | | 1.28 1.92 | | <0.001 | |
| Natural direct effect | 1.40 | 1.18 1.65 |  | 1.41 | | 1.17 1.69 |  | 1.53 | | | 1.26 1.87 | |  | | 1.54 | | | 1.26 1.89 | |  | |
| Natural indirect effect | 1.06 | 1.01 1.10 |  | 1.04 | | 0.97 1.12 |  | 1.02 | | | 0.97 1.07 | |  |  | 1.02 | | | 0.96 1.07 | |  |  |
| Proportion mediated | 14% | | | | 11% | | | | 4% | | | | | | | | 4% | | | | |
|  | **Mediator: Avoidance** | | | | | | | | | **Mediator: Avoidance***** | | | | | | | | | | | |
| Total causal effect | 1.47 | 1.26 1.73 | <0.001 | 1.48 | | 1.25 1.76 | <0.001 | 1.56 | | | 1.27 1.92 | | <0.001 | | 1.57 | | | 1.27 1.94 | | <0.001 | |
| Natural direct effect | 1.38 | 1.17 1.62 |  | 1.40 | | 1.17 1.66 |  | 1.50 | | | 1.22 1.83 | |  | | 1.51 | | | 1.22 1.85 | |  | |
| Natural indirect effect | 1.07 | 1.02 1.12 |  | 1.06 | | 1.02 1.11 |  | 1.04 | | | 0.98 1.11 | |  |  | 1.04 | | | 0.98 1.11 | |  |  |
| Proportion mediated | 17% | | | | 15% | | | | 9% | | | | | | | | 9% | | | | |
|  | **Mediator: Hyper-arousal** | | | | | | | | | **Mediator: Hyper-arousal***** | | | | | | | | | | | |
| Total causal effect | 1.48 | 1.26 1.73 | <0.001 | 1.48 | | 1.25 1.75 | <0.001 | 1.56 | | | 1.27 1.91 | | <0.001 | | 1.57 | | | 1.28 1.93 | | <0.001 | |
| Natural direct effect | 1.39 | 1.18 1.64 |  | 1.41 | | 1.19 1.67 |  | 1.52 | | | 1.23 1.87 | |  | | 1.53 | | | 1.23 1.89 | |  | |
| Natural indirect effect | 1.06 | 1.01 1.11 |  | 1.05 | | 1.01 1.09 |  | 1.03 | | | 0.97 1.09 | |  |  | 1.03 | | | 0.97 1.09 | |  |  |
| Proportion mediated | 15% | | | | 12% | | | | 6% | | | | | | | | 6% | | | | |
|  | **Mediator: Negative beliefs** | | | | | | | | |  | | | | | | | | | | | |
| Total causal effect | 1.56 | 1.28 1.90 | <0.001 | 1.57 | | 1.28 1.93 | <0.001 |  | | |  | |  | |  | | |  | |  | |
| Natural direct effect | 1.46 | 1.21 1.77 |  | 1.47 | | 1.21 1.79 |  |  | | |  | |  | |  | | |  | |  | |
| Natural indirect effect | 1.06 | 0.99 1.14 |  | 1.06 | | 0.99 1.14 |  |  | | |  | |  | |  | | |  | |  | |
| Proportion mediated | 14% | | | | 14% | | | |  | | |  | |  | |  | | |  | |  |

*Study of adolescent PEs, N=2,952; **Study of adult PEs, N=2,492 except for the avoidance and

hyper-arousal clusters where ***N=2,491; ****Confounders adjusted for: sex and family history of

mental health illness

Information on the association between trauma, PTSD clusters and PEs is available on request

S7. Associations between trauma, *PTSD diagnosis* and PEs/psychotic disorder*. Total, direct and indirect effects for PEs/Psychotic disorder: mediator: PTSD diagnosis

| **Exposure** | **Outcome** | **Unadjusted** | | | | | | | **Adjusted**** | | | | | |
| --- | --- | --- | --- | --- | --- | --- | --- | --- | --- | --- | --- | --- | --- | --- |
|  |  | **OR** | | **95%CI** | | **P value** | | **OR** | | | **95%CI** | | **P value** | |
| Trauma  (ages 0-17) | PTSD diagnosis  (age 24) | 1.93 | | 1.61  2.32 | | <0.001 | | 1.94 | | | 1.61 2.34 | | <0.001 | |
| PTSD diagnosis  (age 24) | PEs  (ages 19-24) | 4.89 | | 1.86  12.82 | | <0.001 | | 3.22 | | | 1.67 8.89 | | 0.02 | |
| PTSD diagnosis  (age 24) | Psychotic disorder  (age 24) | 8.20 | | 2.30  29.27 | | 0.001 | | 3.88 | | | 0.98  15.32 | | 0.05 | |
|  | | | | | | | | | | | | | | |
|  | | | **PEs (age 19-24)** | | | | | | | **Psychotic disorder (age 24)** | | | | |
|  |  |  | **Adjusted***** | | | | | | | **Adjusted***** | | | | |
|  |  |  | **OR** | | **95% CI** | | **p** | | **OR** | | | **95% CI** | | **p** |
| Total causal effect | | | 1.58 | | 1.28 1.94 | | <0.001 | | 2.02 | | | 1.46 2.79 | | <0.001 |
| Natural direct effect | | | 1.49 | | 1.22 1.82 | |  | | 1.85 | | | 1.33 2.57 | |  |
| Natural indirect effect | | | 1.06 | | 0.98 1.14 | |  |  | 1.09 | | | 0.95 1.25 | |  |
| Proportion mediated | | | 12% | | | | | | | 12% | | | | |

*N=2,492; ^**^Sex and family history of mental illness adjusted for in all models; Exposure to trauma additionally adjusted for in PTSD diagnosis-PEs/Psychotic disorder regressions; ***Confounders adjusted for: sex and family history of mental illness

S8. Total, direct and indirect effects for hallucinations and delusions

|  | **Hallucinations (12-18)*** | | | | | | | | | **Delusions (12-18)*** | | | | | | | | | **Hallucinations (19-24)**** | | | | | | | |
| --- | --- | --- | --- | --- | --- | --- | --- | --- | --- | --- | --- | --- | --- | --- | --- | --- | --- | --- | --- | --- | --- | --- | --- | --- | --- | --- |
|  | **Unadjusted** | | | | **Adjusted***** | | | | **Unadjusted** | | | | | **Adjusted***** | | | | **Unadjusted** | | | | | **Adjusted***** | | | |
|  | OR | 95% CI | p | OR | | 95% CI | p | OR | | | 95% CI | p | OR | | 95% CI | p | OR | | | 95% CI | p | OR | | 95% CI | p |  |
| Total causal effect | 1.68 | 1.34 2.09 | <0.001 | 1.70 | | 1.35 2.13 | <0.001 | 1.40 | | | 1.16 1.70 | 0.001 | 1.39 | | 1.12 1.71 | 0.002 | 1.47 | | | 1.17 1.85 | 0.001 | 1.48 | | 1.16 1.90 | 0.002 |  |
| Natural direct effect | 1.55 | 1.22 1.97 |  | 1.59 | | 1.25 2.02 |  | 1.32 | | | 1.09 1.61 |  | 1.32 | | 1.07 1.62 |  | 1.39 | | | 1.11 1.74 |  | 1.10 | | 1.23 1.79 |  |  |
| Natural indirect effect | 1.08 | 1.01 1.15 |  | 1.07 | | 1.00 1.13 |  | 1.06 | | | 1.00 1.12 |  | 1.05 | | 1.00 1.10 |  | 1.06 | | | 0.99 1.13 |  | 1.06 | | 0.99 1.13 |  |  |
| Proportion mediated | 15% | | | | 12% | | | | 18% | | | | | 15% | | | | 15% | | | | | 14% | | | |

*Study of adolescent PEs, N=2,952; **Study of adult PEs, N=2,492; ***Confounders adjusted for: sex and family history of mental health illness

Information on the associations between trauma, PTSD and hallucinations/delusions is available on request

References

Daniel, R. M., de Stavola, B. L. and Cousens, S. N. (2011) ‘Gformula: Estimating causal effects in the presence of time-varying confounding or mediation using the g-computation formula’, *Stata Journal*, 11(4), pp. 479–517. doi: 10.1177/1536867x1201100401.
